# A Linkage between Angiogenesis and Inflammation in Neovascular Age-Related Macular Degeneration

**DOI:** 10.3390/cells11213453

**Published:** 2022-11-01

**Authors:** Hanna Heloterä, Kai Kaarniranta

**Affiliations:** 1 Department of Ophthalmology, Institute of Clinical Medicine, University of Eastern Finland, 70211 Kuopio, Finland; 2 Department of Ophthalmology, Kuopio University Hospital, 70210 Kuopio, Finland

**Keywords:** aging, angiogenesis, aggregation, degeneration, inflammation, macula

## Abstract

Age-related macular degeneration (AMD) is the leading cause of visual impairment in the aging population with a limited understanding of its pathogenesis and the number of patients are all the time increasing. AMD is classified into two main forms: dry and neovascular AMD (nAMD). Dry AMD is the most prevalent form (80–90%) of AMD cases. Neovascular AMD (10–20% of AMD cases) is treated with monthly or more sparsely given intravitreal anti-vascular endothelial growth factor inhibitors, but unfortunately, not all patients respond to the current treatments. A clinical hallmark of nAMD is choroidal neovascularization. The progression of AMD is initially characterized by atrophic alterations in the retinal pigment epithelium, as well as the formation of lysosomal lipofuscin and extracellular drusen deposits. Cellular damage caused by chronic oxidative stress, protein aggregation and inflammatory processes may lead to advanced geographic atrophy and/or choroidal neovascularization and fibrosis. Currently, it is not fully known why different AMD phenotypes develop. In this review, we connect angiogenesis and inflammatory regulators in the development of nAMD and discuss therapy challenges and hopes.

## 1. Introduction

**Figure 1:**
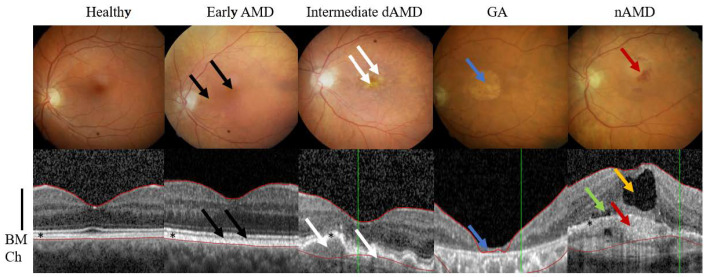
Fundus photographs (upper panel) and optical coherent tomographs (lower panel) from healthy individual, early AMD with pigment mottling (black arrows), intermediate dAMD with drusen (white arrows), GA (blue arrows) and nAMD with choidal neovascularization and hemorrhage (red arrows) that coincide with intra- (yellow arrow) and subretinal (green arrow) fluids. Black column indicates neural retina and asterisk the RPE layer that both are atrophic or missing in GA. Abbreviations: AMD, age-related macular degeneration; BM, Bruch’s membrane; Ch, choriocapillaris; d, dry; GA, geographic atrophy; n, neovascular and RPE, retinal pigment epithelium. Note that BM (red line) is not optically recognized right when pathology increases and thus it starts to wave.

Age related macular degeneration (AMD) is the one of the most common causes of irreversible blindness among the elderly population in developed countries [1]. AMD patient numbers are expected to increase in the future due to aging population and it has been projected that 288 million individuals globally will suffer from AMD in 2040 [2,3]. Increase in global burden of AMD will pose a huge burden on the healthcare system, but also for individuals suffering from these disabling diseases. AMD is classified into two forms according to clinical features; dry AMD and neovascular AMD (nAMD) from which the dry AMD is the most prevalent form, accounting 80–90% of all AMD cases (Figure 1). Dry AMD is also divided into early, intermediate and geographic atrophy (GA) subgroups (Figure 1). GA and nAMD are determined as a late state AMD [4].

Regardless of the higher prevalence of dry AMD, the majority of visual impairments is due to nAMD as the disease has a more aggressive nature and is faster progressing [4]. As the burden of the disease is high, there is a significant need to control progression of these diseases. Effective treatments with novel mode of actions would be needed in order to support patients to maintain their vision, but also treatments that are more durable would be needed to support the healthcare system, which is struggling with resource issues due to high treatment burden. During the last two decades, the management of the nAMD has advanced dramatically due to the arrival of anti-VEGF (vascular endothelial growth factor) therapies, but unfortunately, no such advances have been made in the treatment of dry AMD [4].

**Figure 2:**
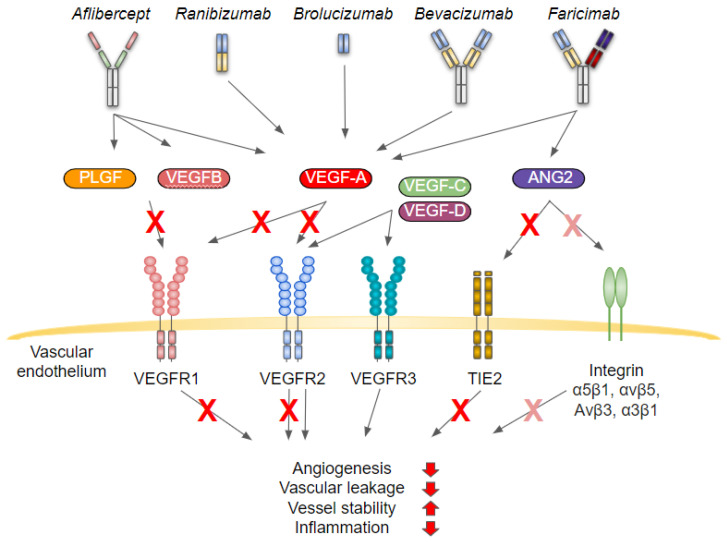
Mechanism of action of anti-VEGF treatments currently used to treat neovascular age related macular edema. Activation of VEGFR2 by VEGF-A plays a major role during angiogenesis, the role of VEGFR1 and VEGFR3 during neovascularization is less pronounced. Binding of ANG2 to TIE2 and Integrin α5β1, αvβ5, α3β1 and αvβ3 will further. All the antiangiogenic agents used to treat age related macular degeneration, binding to VEGF-A and prevent its signaling. Aflibercept, which is a fusion protein, will also bind to PLGF and VEGF-B. Faricimab instead is a bi-specific antibody and will bind to both ANG2 and VEGF-A. Whether faricimab will inhibit binding of ANG2 to integrins is less well characterized than its capability to intervene with ANG2-TIE2 signaling. Both ranibizumab and brolucizumab antibody fragments that inhibit VEGF-A signaling. Brolucizumab is a humanized monoclonal single-chain Fv (scFv) antibody fragment and ranibizumab is a humanized monoclonal antibody fragment. VEGF-C and VEGF-D are ligands for both VEGFR-3 and VEGFR-2 and may mediate pro-angiogenic signals even in the presence of previously mentioned anti-VEGF treatments. Abbreviations: PLGF, placental growth factor; VEGF, vascular endothelial growth factor; ANG, angiopoietin; VEGFR, vascular endothelial growth factor receptor, TIE, tyrosine kinase with Ig and EGF homology domains.

Currently, injection of VEGF inhibitors into the vitreous is the standard of care for nAMD patients, but unfortunately, intravitreal injection of VEGF inhibitors is able to only slow down the disease progression and does not provide a cure [4,5]. Agents including ranibizumab, aflibercept, pegaptanib, brolucizumab and faricimab have been approved by the Food and Drug Administration (FDA) for the treatment of nAMD, but pegaptanib is rarely used as it does not improve visual acuity on average in newly diagnosed nAMD patients (Figure 2) [4,6,7,8,9,10]. In addition to approved treatments, the bevacizumab is commonly used for treatment of nAMD [4,11,12]. All the current treatments act by binding to their target proteins and thus neutralizing their activity. Binding of antibody, antibody fragment or fusion protein to the target ligand will prevent the binding of ligands for their receptor that is expressed on the surface of the target cell. This will further intervene with the downstream signaling of the receptor, which would have been modified due to binding of ligand to the receptor. All the antiangiogenic agents currently used to treat age related macular degeneration will bind to VEGF-A, and therefore, regulate its binding to vascular endothelial growth factor receptor 2 (VEGFR2) and VEGFR1 [11,13]. From which, VEGR2 is presumed to play a major role during angiogenesis providing pro-angiogenic, pro-mitogenic, anti-apoptotic and vascular permeability promoting functions [14]. In addition to VEGF-A, aflibercept also binds to placental growth factor (PLGF) and VEGF-B, which are ligands for VEGFR1 [11]. VEGF family has also third receptor, VEGFR3, with ligands VEGF-C and VEGF-D. Currently approved nAMD treatments do not bind to these ligands, but both ligands are able to bind both VEGFR2 and VEGFR3 [15]. In addition to lymphangiogenesis, signaling through VEGFR3 can also drive angiogenesis, but the pro-angiogenic affect is not as pronounced as with VEGFR2 [16]. Thus, VEGF-C and VEGF-D might be able to compensate some level of VEGF-A blockage and mediate angiogenesis through VEGFR-2 and VEGFR-3 [15,16,17]. Faricimab, instead, can also bind to angiopoietin 2 (ANG2), which is a ligand for TIE2 (tyrosine kinase with Ig and EGF homology domains) and integrin α5β1, α3β1, αvβ5 and αvβ3 [18,19,20]. In addition to ANG2, the ANG1 is also a ligand for TIE2, but mediates opposite effects than ANG2 during neovascularisation. Binding of ANG1 to TIE2 will promote survival and vascular stability. ANG2 instead is a context dependent agonist/antagonist for TIE2 and during neovascularization binding of ANG2 to TIE2 will prevent signaling through TIE2 and will contribute to vascular destabilization, increased inflammation and enhanced angiogenesis [19]. From these agents, ranibizumab and brolucizumab are antibody fragments [13,21]. Ranibizumab is a 48 kDa humanized recombinant antibody Fab (antigen binding fragment), that neutralizes all active forms of VEGF-A [21,22]. Brolucizumab, instead, is a humanized 26 kDa single-chain antibody fragment consisting of variable domains of the monoclonal antibody joined by a flexible linker peptide. Brolucizumab can bind to all VEGF-A isoforms [13,23]. Bevacizumab and faricimab are full length humanized antibodies [24,25]. 149 kDa bevacizumab can bind to all VEGF-A isoforms [24,26]. 150 kDa faricimab instead, is a bispesific monoclonal IgG1 antibody with optimized fragment crystallizable (Fc). Optimization was done to eliminate binding interaction with neonatal Fc and Fc γ receptors, decreasing systemic half-life of the antibody and reducing potential for inflammatory side effects, respectively. As faricimab mode of action is based on ligand binding and neutralization, the effector function of Fc domain is not considered to be needed. Due to heterodimerization of two independent antigen binding domains, faricimab can independently bind both VEGF-A (all isoforms) and ANG2 [25]. Aflibercept instead, is a 97–115 kDa fusion protein consisting of second extracellular Ig domain of VEGFR1 and third Ig domain of VEGFR2 and is fused to the Fc portion of human IgG1. In addition to VEGF-B and PLGF, aflibercept is able to bind all VEGF isoforms [11,27,28,29]. Whereas pegaptinib will only bind and block activity of VEGF165 isoform. Pegaptanib is a 28-base ribonucleic acid aptamer, which is linked to two branched 20 kDa polyethylene glycol moieties [30]. 

Current treatment strategies for nAMD require repeated, frequent intravitreal injections, with the exception of recently approved surgically implantable port delivery devices (Available online: https://www.fda.gov/media/158149/download, accessed on 26 September 2022) [4,6,7,8,9,12,31]. One challenge in treatment is also that subgroups of patients do not even initially respond to current treatments or develop resistance to the current therapies during the treatment [32]. Another issue remains in underlying complications, which are seen with the subset of patients. The most common side effect with intravitreal anti-VEGF inhibitors include, e.g., conjunctival hemorrhage, temporal intraocular pressure increase, uveitis, vitreous floaters and eye pain [6,7,8,10,31]. However, the rarer events are the ones, which cause more concern, such as endophthalmitis and retinal detachments. Moreover, intraocular inflammations and occlusion events are currently under discussion due to elevated levels of these complications have been seen with recently approved brolucimab [8,33]. Considering that current therapies for nAMD may fail for subgroups of patients, there remains a need for novel mode of actions to be introduced for the control of this complex disease. Understanding the mechanisms behind the disease pathogenesis and molecular pathways leading to the development of side effects such as fibrosis will provide tools for future development. As both angiogenesis and low-level inflammation are linked to the nAMD pathogenesis, this article will review the link between angiogenesis and inflammation. Current nAMD treatments are mainly VEGF inhibitors, which by itself gives us a guide that this link should be looked at more closely as VEGF upregulation plays an important role during both angiogenesis and inflammation.

## 2. AMD Phenotypes

**Figure 3:**
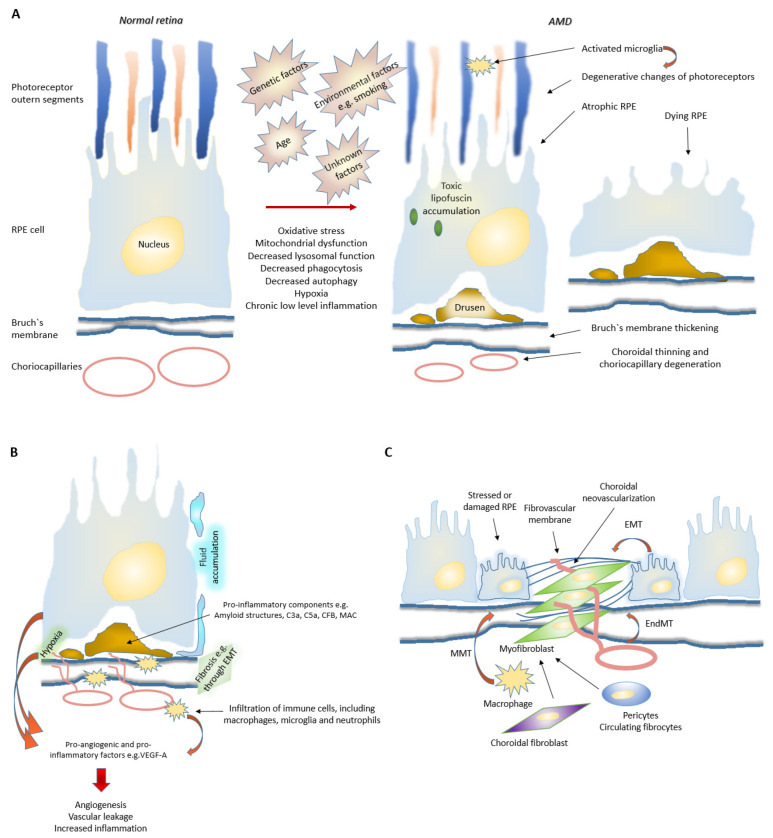
Mechanisms behind age related macular degeneration. (**A**) Age, light exposure, smoking, high-fat diet and unknown factors contribute to elevated oxidative stress, mitochondrial dysfunctions as well as decreased phagocytosis, autophagy and toxin clearance mechanisms in RPE cells. This leads to accumulation of intra- and extra-cellular waste products such as intracellular light absorbing lipofuscin and extracellular drusens. Affected retina may eventually face structural changes such as, RPE degeneration, photoreceptor loss, BrM thickening, choriocapillary degeneration, low-level inflammation and fibrosis. Activated microglia migrate to the outer nuclear layer to remove rod cell debris and, at the same time, may kill adjacent photoreceptors. (**B**) During an angiogenic switch, inflammatory cell recruitment is initiated, and as newly formed vessels are leaky, they contribute to retinal edema, hemorrhage and therefore further potentiate the pathological wound healing response. In nAMD, there exist many mechanisms how neovascularization and low-level inflammation can be stimulated. For example, damaged RPE produces excessive amounts of growth factors, which stimulate vascular growth. In addition, BrM thickening and drusens increase distance of RPE cells from choroidal vasculature and thus increases hypoxia, which in turn will upregulate pro-angiogenic and pro-inflammatory growth factors. Interestingly, drusens also consist of pro-inflammatory components, such as amyloid structures and C3a. Furthermore, accumulating immune cells may secrete inflammatory cytokines and angiogenic growth factors and therefore support the disease progression. (**C**) In nAMD, fibrosis is the product of defective and excessive wound healing response. It is believed that pro-inflammatory cytokines can promote differentiation and activation of matrix-producing myofibroblasts (e.g., EndMT and EMT). Multiple cell types, including fibroblast, fibrocytes, macrophages, RPE cells and endothelial cells, may potentially participate in this process. Mesenchymal cells in subretinal fibrotic lesions can for example, originate from the retinal pigment epithelium and/or choroidal endothelial cells through EMT and EndMT. In addition, macrophages are able to transdifferentiate to myofibroblasts through MMT. RPE cells and infiltrating macrophages are believed to be a major source of these cytokines. Moreover, hypoxia may promote EMT in RPE cells. Abbreviations: RPE, retinal pigment epithelium; EMT, epithelial to mesenchymal transition; C3a, complement component 3a; C5a, complement component 5a; CFB, complement factor B; MAC, membrane attack complex; VEGF-A, vascular endothelial growth factor A; EMT, epithelial–mesenchymal transition; EndMT, endothelial–mesenchymal transition; MMT, macrophage-mesenchyman-transition.

AMD is a complex and multifactorial disease with linked etiologies such as age, genetic factors, cardiovascular diseases, smoking and unhealthy diet [34,35,36,37]. It is a progressive disease, where degeneration of the retinal pigment epithelium (RPE) results in the death of photoreceptors, which in turn leads to a loss of central vision. Aging RPE faces oxidative stress, a factor that together with deteriorating functionality, e.g., decreased intracellular recycling and degradation of proteins that induces inflammation (Figure 3) [36,37,38]. Severity of the symptoms guides the disease classification and AMD can be divided into early, intermediate, and advanced forms (Figure 1) [4]. 

Early AMD is usually asymptomatic, even though accumulation of intracellular lipofuscin in the RPE and small extracellular drusens between RPE cells and Bruch’s membrane can be clinically detected (Figure 1). Instead, intermediate AMD is characterized by an accumulation of multiple medium-sized or at least one big drusen (>125 μm). The advanced AMD is subdivided into two types of GA and nAMD. Term GA is used to describe areas where RPE cells and secondarily photoreceptors have completely damaged or died. Early, intermediate and GA forms consider only dry AMD, while choroidal neovascularization is a clinical hallmark in nAMD. Individuals with advanced AMD often have serious changes in their near and far vision. A functional degeneration of the RPE results in damage of photoreceptors and impaired maintenance of the sensory retina that led to loss of accurate vision and color detection. Thus, AMD greatly impairs the ability of an elderly patient to lead an independent life [4].

## 3. Evolution of AMD

### 3.1. Disturbed Proteostasis in AMD

The exact mechanism behind RPE degeneration and the onset and progression of AMD are not fully understood. However, dysfunction of many cellular processes, such as mitochondrial dysfunction, increased reactive oxygen species (ROS) production and protein aggregation, impaired autophagy and chronic inflammation have been linked to the development of AMD (Figure 3) [35,36,37,38,39,40]. Cellular mechanisms have been described in a Kaarniranta et al. recent publication [39]. For example, emerging evidence suggests that an excess of intracellular ROS production decreasing autophagy plays an important role during AMD development and progression. Autophagy in late AMD occurs at a lower rate than in the early stages of the disease. In general, autophagy is a self-clearance pathway that removes dysfunctional cellular components through lysosomal dependent mechanism and thus protects retinal cells from e.g., oxidative stress induced insults [36,37,39]. Interestingly, hypoxia, which is also a strong driver of angiogenesis, has been shown to induce autophagy, which in turn might act as a survival mechanism for hypoxic cells through recycling of cellular constituents [41,42]. Dysfunctional lysosomal clearance and accumulation of waste materials play key roles in cellular levels during AMD development. Autophagy is especially important for normal RPE homeostasis as RPE cells have undergone terminal differentiation and do not divide anymore or divide only rarely. One of the key roles of RPE cells is phagocytosis of lipid-rich photoreceptor outer segments (POS). POS are degraded in lysosomes. This process is called heterophagy that should be differentiated from autophagy, although lysosomes are regulating both processes. Accumulated lipofuscin during AMD development disturbs both processes heterophagy and autophagy [43]. Oxidative stress is known to induce autophagic flux in RPE cells, but chronic oxidative stress seems to lead to increased lysosomal lipofuscin but decreased lysosomal activity and autophagy. While these processes play an important role in the homeostasis of RPE cells, their impairment can lead to detrimental accumulation of damaged organelles and abnormal or toxic proteins [36,39]. Disturbed proteostasis is clearly demonstrated by the accumulation of lysosomal lipofuscin in the RPE and extracellular drusen deposits between the RPE and choriocapillaris [35]. It is shown that lysosomal degradative functions can be further inhibited in RPE cells by a lipofuscin, and accumulation of lipofuscin in the RPE is a sign of cellular senescence in AMD. Intralysosomal accumulation of toxic lipofuscin can also sensitize lysosomes to visible light, oxidative stress, jeopardize lysosomal stability and lead to cellular apoptosis [44]. Drusens, instead, are yellowish lipid and protein rich deposits, whose accumulation will eventually lead to functional loss of the retinal photoreceptors (Figure 1). It is believed that RPE cells are the origin of numerous components found in drusen deposits. Drusen deposition is considered as the hallmark of AMD, and the accumulation of large drusens increases an individual’s risk of developing advanced AMD. It has been shown that chronic low-level inflammation, elevated oxidative processes, stressed autophagy, complement activation, changes in choriocapillaries and increased exo- and transcytosis in RPE cells play roles in drusen formation (Figure 3). Interestingly, when formed, drusens can probably escalate disease progression as isolated drusen material has also been proven to be pro-inflammatory. Drusens can enhance inflammation for example through amyloid structures, and inflammasome pathways [35,45,46,47]. In addition, C3a, C5a, CFB, and membrane attack complex (MAC) complexes found in drusens have been linked to increased expression of VEGF and the formation of choroidal neovascularization (CNV, Figure 3) [48,49,50,51,52,53]. 

### 3.2. Role of Choriocapillaris in nAMD Development

**Figure 4:**
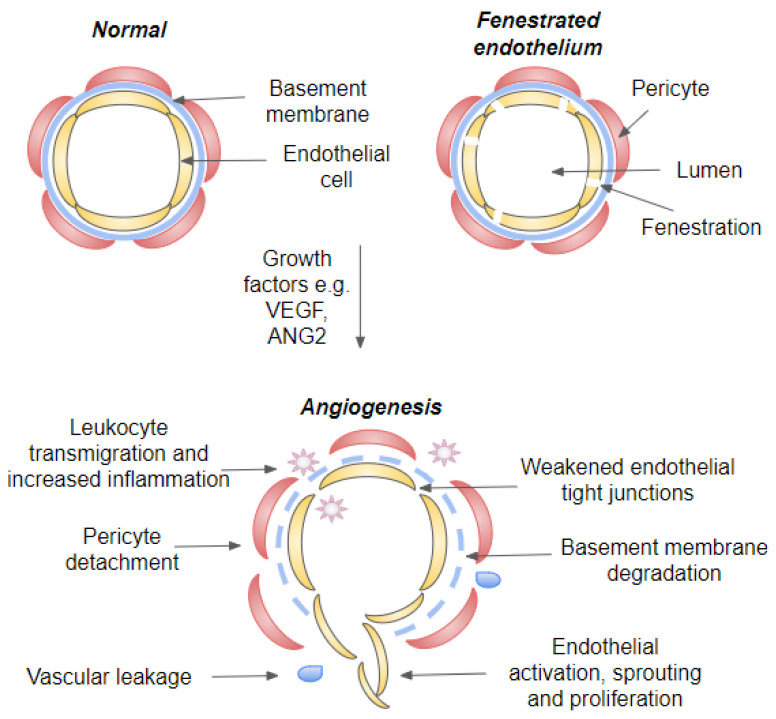
Illustration of a blood vessel in quiescent and angiogenic state. Cross-section of a capillary, fenestrated capillary and blood vessel during neovascularization. During vascular homeostasis, endothelial cells are connected by tight junctions and covered by both basement membrane and pericytes. Growth factors such as VEGF-A and ANG2 induce angiogenic switch that will lead to weakening of endothelial tight junctions, pericyte dropout and initiation of angiogenesis. Endothelial destabilization will further lead to vascular leakage and leukocyte transmigration. Abbreviations: VEGF, vascular endothelial growth factor; ANG, angiopoietin.

It should be noted that drusens together with age and disease related Bruch’s membrane (BrM) thickening increase the barrier between the choriocapillaris and the highly metabolically active outer retina, resulting in critically reduced metabolite delivery (Figure 3) [1,54]. Choriocapillaries are the innermost region of choroid and are located just under the BrM and RPE. In addition, they have a rather large diameter (20–50 μm) and fenestrated endothelium to support the delivery of nutrients and export of waste products (Figure 4). Under normal conditions, 85–90% of oxygen demand of outer retina is delivered by the choriocapillaris, while the remaining 10–15% is obtained from the deep retinal capillary plexus. For the entire retina, the choroid is responsible ~60 % of oxygen and ~75% nutrient supply. In the outer retina, the majority of oxygen is consumed in the photoreceptor inner segment where densely packed mitochondria produce ATP via aerobic respiration. To respond to the retina’s high rate of oxygen consumption, the choroid is perfused at a high rate and the drop in blood oxygen concentration along the vascular plexus to just 1% between arterioles and venules [47,55]. Thus, the proper functioning of choriocapillaries is critical to support RPE and the rest of the retina. It is observed that during aging and early stages of AMD development, choroidal blood flow drops and both choroid and choriocapillaries become thinner. This degeneration includes loss of capillaries and reduction in endothelial cell fenestrations. It is believed that these changes can precede RPE damage. Interestingly, it is reported that decreased choriocapillary density correlates with increased drusen formation. This finding suggests that proper functioning of choriocapillaries is important for preventing drusen formation. In advance nAMD, reports from the choroidal thickness are controversial, which may be due to underlying neovascularization. Interestingly, regions with choroidal neovascularization are often surrounded by localized nonperfused choriocapillary regions and it can be speculated that hypoxia may further regulate the location of neovascular lesion development [47,55].

### 3.3. Senescence in AMD

During AMD progression, decreased autophagy is also associated with cellular senescence and senescence-associated secretory phenotype is associated with the release of ROS, selective growth factors, and inflammatory cytokines, chemokines and proteases. Senescent cells also promote the senescence of surrounding cells’ secretory phenotype, which can contribute to the maintenance of a chronic state of low-grade inflammation in tissues and organs. They are also apoptosis resistant, failing to enter programmed cell death and rather aggregate instead. Therefore, they may regulate drusen biogenesis and advanced GA and nAMD development [56,57]. Term immunosenescence is used to describe altered immune functions during aging. Aging alters the functions of the immune system so that it no longer resembles the immune system of young individuals. One of the hallmarks of immunosenescence is lingering low-grade inflammation that is present also in AMD. Human immune system can efficiently fight acute infections when we are young but is not so efficient for fighting chronic stimulus. Thus, chronic situations lead to an accumulation of pro-inflammatory cytokines and acute phase proteins. Increased oxidative stress, reduced proteostasis, and increasing dysfunctionality are just some of the stress factors that can induce inflammation in aged RPE cells [58,59]. To date, it is not understood why one develops GA, while another has nAMD under chronic inflammation. 

### 3.4. Fibrosis in nAMD

The transition from early to advanced AMD shares many features with a defective wound healing response resulting from underlying oxidative stress, degeneration and chronic inflammation (Figure 3). Subretinal fibrosis is a characteristic of the end-stage of AMD, resulting in a permanent vision loss. Wound healing will occur when injured tissue activates recruitment and activation of inflammatory cells and fibroblasts. In AMD, fibrosis is the product of defective and excessive wound healing response, which has unique characteristics as in nAMD it can originate from pre-existing neovascular membrane [60,61]. Multiple cell types, including fibroblast, fibrocytes, macrophages, RPE cells and endothelial cells, may potentially participate to this process. Matrix-producing mesenchymal cells in subretinal fibrotic lesions can for example originate from the retinal pigment epithelium and/or choroidal endothelial cells through epithelial–mesenchymal transition (EMT, Figure 3) and endothelial–mesenchymal transition (EndMT). Moreover, macrophages are able to transdifferentiate to myofibroblasts through macrophage-mesenchyman-transition (MMT). Majority of mesenchymal cells present in fibrotic lesions are myofibroblasts, which are not normally present in adult tissues [60,61]. It is believed that pro-inflammatory cytokines can promote differentiation and activation of myofibroblasts (e.g., EndMT and EMT). RPE cells and infiltrating macrophages are believed to be a major source of these cytokines. During EMT, EndMT and MMT cells experience several biochemical and morphological changes, they, for example, become more motile and adaptable. They lose cell-to-cell contacts and cell polarity, and further disengage from their basal surface and basement membrane in order to migrate and produce extracellular matrix components. For example, during EMT, the resulting mesenchymal cell leaves the epithelial layer and migrates into the retinal and subretinal RPE space and promotes fibrosis. Fibrosis in nAMD is considered to originate from neovascular membranes and to be a consequence of a fibrovascular scarring resulting from inflammation and hypoxia-driven angiogenesis [60,61,62,63]. Fibrosis may in some circumstances restore the protective barrier but can also progressively remodel and destroy normal tissue, leading to contracture and distortion of tissue architecture. As retinal visual function is built on highly organized anatomical layers and tightly coordinated cellular interactions, subretinal fibrosis will lead to a profound and often irreversible visual impairment. Currently, the pathogenetic mechanisms of subretinal fibrosis are poorly understood, and there is no therapy that would prevent excessive subretinal fibrosis [61]. It is hypothesized that during nAMD progression, RPE cells might avoid cell death and escape from the stressful microenvironment and oxidative insult via EMT, but the development of fibrosis is also linked to angiogenesis [57,60,64]. During an angiogenic switch, inflammatory cell recruitment is initiated, and as newly formed vessels are leaky. They contribute to retinal edema, hemorrhage and therefore further potentiate the pathological wound healing response (Figure 4) [65,66,67,68]. 

Considering the wide array of cellular processes involved in nAMD development, the nAMD can`t be recognized only as an exudative vascular disease, but we should also consider that a chronic low-grade inflammation in the retina and choroid is also associated with pathogenesis. Elevated expression of local and systemic biomarkers indicates that chronic inflammation is involved in the pathogenesis of both dAMD and nAMD. In nAMD, the RPE produces excessive amounts of growth factors, which stimulate vascular growth [36]. Of these factors, VEGF is most well characterized in AMD [68]. Increasing levels of growth factors will lead to choroidal CNV, a growth of abnormal blood vessels from the choroid extending into the avascular RPE and sub-retinal regions [36]. Newly formed abnormal blood vessels are leaky, which in turn causes swelling to surrounding tissues and an acute vision loss. There are potentially many sources for these growth factors in retina. For example, it has been shown that RPE cells can produce VEGF-A via e.g., oxidative stress induced senescence and complement activation (Figure 3) [48,69,70].

## 4. Angiogenesis and Inflammation

Blood vessel formation can be divided into vasculogenesis and angiogenesis. Vasculogenesis involves a de novo development of blood vessels and differentiations of endothelial cells from angioblasts and occurs mainly during embryogenesis. Whereas angiogenesis is a development of vascular capillaries from pre-existing blood vessels and is responsible for the further modeling of the vascular networks [71]. It is a fundamental process during development and tissue regeneration. Angiogenesis is stimulated when hypoxic, diseased, or injured tissues produce and release factors that promote angiogenesis (Figure 3). These proangiogenic growth factors stimulate the migration and proliferation of endothelial cells from preexisting vessels and, subsequently, the formation of capillary tubes and the recruitment of other cell types to generate and stabilize new blood vessels (Figure 4) [71].

Angiogenesis and inflammation are seemingly two different processes but are closely linked together as especially angiogenesis occurring in adult organisms is often linked to the inflammation [66]. Inflammation is a cellular response to factors that challenge the homeostasis of cells or tissues and is intended to eliminate foreign or damaged material. At the beginning of an inflammatory response, foreign or damaged material becomes sensed by various pattern recognition receptors (PRRs). The ligand recognition process activates intracellular signaling pathways, resulting in the production of numerous proinflammatory mediators [36,72,73,74]. Inflammation can destroy or inactivate invading pathogens, remove waste and debris, and permit restoration of normal function, through either resolution or repair. The goal of the inflammatory process is to repair damaged tissue in order to restore the typical tissue architecture, thus maintaining cellular/tissue homeostasis. After resolutions of inflammation, tissue structure should be normal, whereas repair leads to a functional, but morphologically altered organ. During acute inflammation, tissue damage is followed by resolution, whereas in chronic inflammation, damage and repair continue simultaneously. The initial inflammatory response is often acute, and depending on the circumstances, may evolve into chronic inflammation. Although inflammation is usually beneficial to the organism, it may also lead to tissue damage, resulting from the escalation of chronic inflammation [36,72,73,74]. Interestingly, retinal autoantibodies have been found in a great majority of patients with early stage and late stage of AMD, thus they might play a role in disease progression towards GA and nAMD [75,76,77].

### 4.1. Inflammatory Signaling Cascades

A number of signals, ranging from microbes and other foreign material to mechanical tissue injury and autoantigens, can stimulate inflammation. Although it is a crucial survival mechanism, prolonged inflammation is detrimental and plays a role in numerous chronic age-related diseases [66,78]. Inflamed tissues are characterized by a hypoxia and immune cell infiltration, a process that will result as an upregulation of molecular and cellular mechanisms that will regulate angiogenesis (Figure 3 and Figure 4). Inflammation-associated angiogenesis is linked to several pathophysiological processes, such as cancer and scar formation. In addition, the role of chronic inflammation has been emerging in conditions such as autoimmune diseases and neurodegenerative diseases [66,78]. During acute inflammation, fluid and immune cells accumulate at the site of injury due to changes in small blood vessel integrity. Cells, which are damaged by, e.g., cellular stress or infectious agents, expose molecules called as alarmins or damage associated molecular patterns (DAMP) [36,73]. These molecules become sensed by a variety of cells that express PRRs, which will induce amplification of immune response. During this process, inflammasome and nuclear factor kappa B (NF-κB) signaling pathways become activated and a number of proinflammatory cytokines and other inflammatory mediator will be released. The cytokines and inflammatory mediators include molecules such as VEGF, IL-1α, IL-1β, IL-8 and TNF-α, as well as histidine, thrombin and fibrinogen. These molecules activate endothelial cells, induce vasodilation and increase vascular permeability, which will further facilitate immune cell transmigration to eliminate the aggressive agent [36,73]. Endothelial cell activation is characterized by increased expression of leukocyte adhesion molecules, cytokines, growth factors, HLA molecules and will lead to changes in endothelial cell junctions and surrounding pericytes (Figure 4). Vascular phenotype will further change from antithrombotic to prothrombotic, in order to prevent the spreading of a potential pathogen and platelets participate in the coagulation process in order to prevent blood loss from damaged vessels. Subsequently, vasodilation occurs, and permeability of blood vessels increases, allowing inflammatory mediators and immune response cells, including leukocytes and monocytes/macrophages, to infiltrate damaged tissue. Which will further modify the microenvironment in the retina [70,73,78,79]. Intriguingly, it has been shown that patients with nAMD have increased levels on circulating CD11b+ monocytes and accumulations of macrophages and mononuclear cells around choroidal neovascular lesions (Figure 3). In experimental settings, CD11+ cells have been also demonstrated to secrete inflammatory cytokines and angiogenic growth factors and therefore support for disease progression. In fact, Subhi et al., recently demonstrated that levels of circulating CD11+ monocytes correlate with amount of needed anti-VEGF injections underlying the involvement of an immune component in nAMD [80].

### 4.2. Blood-Retinal-Barriers

Macular edema results due to vascular hyperpermeability and is identified by swelling of the central portion of the human retina, and is associated with increased retinal thickness. It can be defined as an excess of fluid within the retinal tissue (Figure 3). Interstitial spaces of the retina are normally relatively dry as free leakage of fluid and protein from the macular vasculature is prevented by the blood-retinal-barrier (BRB). BRB can be divided into outer BRB (oBRB) and inner BRB (iBRB), which control the passage of substances in outer and inner retina, respectively. Outer BRB includes the choroid, Bruch`s membrane and RPE [81]. Vasculature of oBRB includes choriocapillaries, which are maintained by the VEGF produced from the basolateral side of the RPE cells and actively supply nutrients as well as remove waste products from outer retinal layers [70,82]. As choriocapillaries are fenestrated, they do not provide barrier by themself, but barrier is formed by Bruch`s membrane and RPE [70,81]. Interestingly, ultrastructure of choriocapillaries is further polarized as fenestrations locate on the retinal side of the vessels and pericytes are found from the outer wall facing sclera. This structure further facilitates efficient movement of macromolecules between retina and choroid [83]. Paradoxically, fenestrated vasculature is dependent on constant VEGF supply [84], but choriocapillaries in human still seem to tolerate long-term treatment with anti-VEGF inhibitors in ocular conditions. As a comparison, the kidneys have also fenestrated vasculature, and it has been reported that cancer treatment with anti-VEGF treatments will cause changes in kidney function [85,86,87]. Furthermore, it has been demonstrated by mouse models, that maintenance of horiocapillaris is dependent on constant VEGF supply [82,84]. However, interestingly, long-term anti-VEGF usage in AMD patients has been reported to result only as slightly reduced choriocapillary density and clinical significance of this finding remains still unknown. Nevertheless, studies done with diabetic macular edema patients have failed to show similar findings [88]. It is not yet fully understood why fenestrated choriocapillaris seems to tolerate VEGF-inhibition, but there may be many possible mechanisms for that. On one hand, it may be that concentrations of anti-VEGF reaching choriocapillaris after intravitreal injection may not be high enough to cause deleterious effects and relatively short half-life of anti-VEGF inhibitors in vitreous may further support this effect. On the other hand, tissue microenvironment may also be able to compensate inhibitory effect by increasing VEGF production or by other growth factors. It has been also demonstrated that also choroidal endothelial cells may express VEGF [89], and therefore one option is that intracrine or autocrine VEGF signaling could be enough to support the maintenance of fenestrated choriocapillaris in humans.

In contrast to oBRB, the vasculature of iBRB includes retinal vasculature that supplies inner retina and originates from the central artery. The retinal capillaries are different from most other peripheral capillaries, as they are almost impermeable to proteins, electrolytes, and water-soluble nonelectrolytes. They are surrounded by thick basement membrane and tight junctions join endothelial cells. Today, the BRB is understood to play a key role in retinal function in both health and disease. AMD is characterized by alterations in Bruch`s membrane, RPE and choroidal capillaries, which contribute to the breakdown of outer BRB. For example, Bruch`s membrane goes through alterations, including thickening and calcification, which lead to reduced permeability and elasticity of Bruch`s membrane. As a consequence, gasses, nutrients and waste products do not pass Bruch`s membrane properly, which may damage the retina and accelerate, e.g., nAMD progression due to hypoxia [70,81]. Interestingly, involvement of inner BRB breakdown in dAMD development has been reported recently [90]. The extracellular fluid of the retina is regulated by vascular endothelium, which consists of adjacent endothelial cells connecting with each other by lateral cell-cell junctions. Forming junctions are called tight-junctions that involve specific molecules, claudins and occludins, which form a zipper-like structure between cells that controls the paracellular passage of ions and solutes [70,78,81]. Although the molecules extravasating from blood vessels can reach the extravascular space by transcellular transport directly through the endothelial cell cytoplasm, the majority of available data indicate that the most frequent pathologic mechanism is breakdown of the interendothelial junctional complexes. This route is for example, primarily involved in cell migration. It is important to realize that one of the mechanisms of how intravitreal anti-VEGFs works is that they stabilize the BRB and correct abnormal permeability in retinal disease [70,78]. Interestingly, in addition to VEGF inhibition, recently approved faricimab will inhibit also ANG2, which is upregulated in retinal/choroidal diseases and contributes to vascular instability and inflammation (Figure 2) [19].

### 4.3. Inflammatory Cell Recruitment

Already 1985 Penfold et al., described the involvement of immunocompetent cells in early, intermediate and late stage of AMD. They suggested that macrophages, fibroblast, lymphocytes and mast cells play a role in neovascularization, atrophy of RPE and Bruch`s membrane breakdown [91]. During the inflammation process, leukocytes sense the chemokine gradient originating from the inflamed tissue and begin to make contact with the adhesion molecules expressed by endothelial cells to permit their tighter binding to the vascular endothelium. Finally, leukocytes leave the circulation by following the chemokine gradient and move towards the damaged tissue, where they become activated [72]. Studies done with an inducible model of photoreceptor death in mice showed infiltration of 12 distinct subpopulations of microglia, monocytes and macrophages [92]. Previously mentioned study did not identify markers for neutrophils, but infiltration of neutrophils has been described from early AMD patient samples and studies done with mouse models suggested the role of neutrophils in retinal degenerations [92,93]. Interestingly, neutrophil depletion in CCR2 knockout mice resulted in a reduction in choroidal neovascularization after laser photocoagulation [94]. Activation of immune cells at the target tissue will involve a variety of cellular processes. Neutrophils, for example, can try to eradicate the cause of inflammation by releasing toxic content such as reactive oxygen species. Monocytes, instead, can differentiate into macrophages and dendritic cells according to the local conditions [72]. Macrophages are very adaptive cells changing their phenotype and functions depending on the environment. They are resident cells in choroid also under physiological conditions when they remove debris and dying cells from the tissue. It has been hypothesized that excessive activation of debris and byproducts products in AMD will exceed the clearance capacity of resident macrophages and microglial cells and give rise to chronic inflammation and immune cell infiltration [95]. Indeed, it has been shown that in postmortem eyes elevated levels of IBA1^+^ (ionized calcium-binding adapter molecule 1) macrophages can be observed in submacular and paramacular choroid in early and intermediate AMD. Whereas the amount of HLA-DR^+^ (human leukocyte antigen-antigen D-related) macrophages is increased during all stages of AMD. HLA-DR^+^ macrophages in AMD samples are also more rounded and smaller in size, which signals for their activation. In this study, an increase in IBA1^+^ and HLA-DR^+^ macrophages associated with the appearance of choroidal neovascularization in nAMD [96]. To complement these finding, presence of soft drusens and continuous thick basal laminar deposits has been shown to be associated with recruitment of magrophages. In the same study, a high number of Bruch`s membrane associated magrophages was found both in eyes with subclinical CNV and eyes with active CNV [97]. In addition, macrophages derived from peripheral blood monocytes of nAMD patients showed a proangiogenic and inflammatory protein expression profiles in classic (M[IFNγ and LPS]) and alternative (M[IL-4 and IL-13]) polarized macrophages. Same study showed that activated macrophages were proangiogenic both ex vivo and in vivo [98]. To support these finding, it has been also suggested that TNFα and IL-1β produced by macrophages can upregulate production of VEGF in RPE cells [99]. Furthermore, it has been demonstrated that macrophages express VEGF and tissue factor (TF) during active CNV. As TF is involved in fibrin formation it may promote fibrosis and provide scaffold for CNV formation [100]. As fibrosis plays detrimental role in advanced AMD, it is interesting to note that macrophages can act as a fibrosis mediator in different ways e.g., while the tendency of macrophages to secrete extracellular matrix components helps in wound healing, in chronic conditions it can lead to pathological fibrosis [36,101,102].

The role of the immune system during acute tissue damage and defense against foreign antigens has been well characterized, but its role for development of chronic age-related conditions should be more deeply understood. During the development of AMD, aging and oxidative stress will lead to the accumulation of waste product, which will endure para-inflammation to repair and remodel tissue through activation of microglial, macrophage and complement system [36,95]. Neuroinflammation is always accompanied by microglial activation with the release of inflammatory mediators and phagocytosis. Microglia play a role both in innate as well as adaptive immunity and are resident cells in central nervous system. Under physiological conditions, they are in inactive state and release neuroprotective as well as anti-inflammatory factors. Instead, when damage occurs in the central nervous system, they can mediate both protective and harmful actions. Beneficial actions limit the further injury and include removal of waste and degenerated cells as well as the secretion of neuronal survival factors. However, microglia can also promote persistent inflammation and recruit additional inflammatory cells [36,95]. It has been shown that during AMD, migroglial accumulates in the subretinal spaces at the sites of choroidal neovascularization and retinal degeneration. In addition, studies done with CX3CR1 -/- mice demonstrated that subretinal microglial accumulation is associated with retinal degenerations and observed drusen like structures were lipid bloated microglial cells. This study also revealed that activated subretinal microglials express VEGF-A, exacerbate neovascularization and retinal degeneration [103]. It has been also shown that activated microglia are present in outer nuclear layers during rod cell death in AMD. This finding leads to a hypothesis that activated microglia migrate to the outer nuclear layer to remove rod cell debris and at the same time may kill adjacent photoreceptors [104].

During inflammation, the arrival of increasing numbers of immune cells exacerbates the inflammatory response and induces chronic inflammation. It is not only white blood cells that enter the inflamed tissue but also fluids and various plasma proteins gain access to the damaged tissue (Figure 3 and Figure 4). Extravasated plasma proteins such as fibrinogen may further stimulate neovascularization. Inflammatory cells, including macrophages, lymphocytes, mast cells, and fibroblasts, and the angiogenic factors they produce, can also stimulate vessel growth [36,72]. Many proinflammatory cytokines, such as tumor necrosis factor (TNF)-α, IL-6, IL-1β, IL-8 and VEGF, have both angiogenic and proinflammatory activity [14,105,106,107,108]. All the previously mentioned cytokines have been also linked to development of AMD [14,105,109,110,111,112,113]. Upregulation of these factors activates the endothelial cells and promotes angiogenesis (Figure 4). During neovascularization, endothelial cells proliferate and migrate to form new capillaries, contributing to restoring nutrient levels and facilitating immune cell migration. At the beginning of neovascularization, macrophages can for example, promote the degradation of extracellular matrix via matrix metalloproteinases. Proteolytic enzymes released by macrophages allow endothelial cell migration and recruitment of growth factors to form new capillaries. Thereafter, macrophages release several proangiogenic cytokines, which directly or indirectly stimulate endothelial proliferation, migration, or tube formation. In this changing microenvironment, the immune cells gradually modify their cytokine profile, sustaining the inflammatory environment. Chronic inflammation is characterized by tissue infiltration by macrophages, monocytes and lymphocytes, as well as proliferation of blood vessels and connective tissue [66,73,74].

### 4.4. Effects of Hypoxia

Inflammation can promote angiogenesis in several ways, e.g., inflammatory tissue is often hypoxic, and hypoxia can induce angiogenesis through upregulation of factors such as VEGF [72,73]. Normally, mammalian cells are dependent on the oxygen and nutrients so they locate within 100 to 200 μm of blood vessels [114], but if tissue environment faces changes e.g., due to inflammation induced swelling, the existing blood vessels may not be able to provide enough oxygen to the tissue and the hypoxia may occur. Interestingly, hypoxia is one of the factors that can be regarded as a common nominator for both inflammation and angiogenesis, while reduced choroidal perfusion and hypoxia have been also linked to the development of the AMD (Figure 3). Retina has a high metabolic rate and requires a constant supply of nutrients, along with the exclusion of waste materials and therefore an adequate blood supply is needed to fill the requirements. Thus, it is expected that changes such as drusens, age-related thickening of the Bruch`s membrane or swelling in the tissue microenvironment will easily promote hypoxia, inflammation and lead to the formation of new leaky blood vessels [115].

Hypoxia inducible factors (HIFs) are major oxygen sensors and a transcription factor that are activated during hypoxia. Especially HIF1α regulates the number of growth factors that are associated with angiogenesis and inflammation [115]. Interestingly, expression of HIF1α and HIF2α has been observed in human choroidal neovascular membranes [116,117]. Furthermore, it has been it has been shown with mouse models that *Hif1*α (hypoxia-inducible factor) deletion in retinal layers will affect development of the vascular plexus in corresponding retinal layers [118,119]. To supplement these findings, *Hif1*α expression promoted neovascularization in the preclinical mouse model and was associated with upregulation of VEGF expression [120]. Together, these results suggest that HIF1α may be directly or indirectly required for the development of the retinal vasculature. Intriguingly, studies done with human RPE cell 2D and 3D cultures suggest that HIF1α and TGF-β2 can synergistically induce EMT of RPE cells [121].

## 5. Concluding Remarks

This review was aimed at establishing the relationship between inflammation and angiogenesis occurring during nAMD development. Purpose was to summarize mechanisms during endothelial activation, which leads to increased vascular permeability and the initiation of pathological angiogenesis. nAMD is an exudative ocular disease with low-level chronic inflammation and angiogenesis linked to its pathogenesis. Currently, there is no cure to AMD, and changes to the tissue microenvironment are already significant when the first symptoms appear. Further investigation of the association between inflammation and angiogenesis in nAMD is required for a better understanding of the underlying molecular events in these processes. In the future, selected molecules may be useful as therapeutic targets for the reprogramming of homeostasis in order to be able to reverse nAMD progression, avoid resistance to current treatments, lead to improved clinical outcomes and eliminate high injection burden. Rather than completely inhibiting CNV in nAMD, it might be beneficial to develop treatments that will have multiple targets. One approach would be to aim to stabilize damaged and leaky vessel, which could lead to a reduced leakage, inflammations and fibrosis. Alternatively, reduction in intracellular inflammation in conjunction with the prevention of RPE and photoreceptor loss as well as inhibition of fibrosis may provide opportunities for developing novel therapy options for nAMD. Ultimately, in order to aim for cure, we should find ways to reverse underlying condition, or the hypoxia/ischemia caused by drusens, Bruch`s membrane thickening and choroidal degradation, which are driving angiogenesis. Moreover, identification of novel accurate biomarkers could also help to clarify the mechanisms behind nAMD, as well as helping to monitor the response to therapy and facilitate early detection of the disease. Recent research has identified many molecular targets other than anti-VEGFs, as well as alternative drug delivery routes, offering potential new avenues for treating nAMD. It will be interesting to see if, for example, combination of VEGF and ANG2 inhibition translates to reduced fibrosis in clinical use. It could be presumed that employing multiple targeted approaches to treat nAMD could yield better results than targeting just a single pathway.

## Data Availability

Not applicable here.
